# Specialized Pro-Resolving Mediators as Potential Regulators of Inflammatory Macrophage Responses in COVID-19

**DOI:** 10.3389/fimmu.2021.632238

**Published:** 2021-02-24

**Authors:** Maria G. Balta, Evangelos Papathanasiou, Panagiotis F. Christopoulos

**Affiliations:** ^1^ The CrossTalk Group, Institute of Oral Biology, University of Oslo, Oslo, Norway; ^2^ Department of Periodontology, Tufts University School of Dental Medicine, Boston, MA, United States; ^3^ Center for Clinical and Translational Research, Forsyth Institute, Cambridge, MA, United States; ^4^ Department of Pathology, Oslo University Hospital, Oslo, Norway

**Keywords:** resolution of inflammation, lipid mediators/specialized pro-resolving mediators (SPMs), cytokine storm, macrophage activation syndrome (MAS), severe acute respiratory syndrome corona virus (SARS-CoV2)

## Abstract

The recent outbreak of SARS-CoV2 has emerged as one of the biggest pandemics of our century, with outrageous health, social and economic consequences globally. Macrophages may lay in the center of COVID-19 pathogenesis and lethality and treatment of the macrophage-induced cytokine storm has emerged as essential. Specialized pro-resolving mediators (SPMs) hold strong therapeutic potentials in the management of COVID-19 as they can regulate macrophage infiltration and cytokine production but also promote a pro-resolving macrophage phenotype. In this review, we discuss the homeostatic functions of SPMs acting directly on macrophages on various levels, towards the resolution of inflammation. Moreover, we address the molecular events that link the lipid mediators with COVID-19 severity and discuss the clinical potentials of SPMs in COVID-19 immunotherapeutics.

## Introduction

The recent outbreak of the corona virus disease 2019 (COVID-19) from Wuhan, China (1) has evolved to one of the biggest global pandemics of the 21st century. The infectious virus named by the World Health Organization as severe acute respiratory syndrome corona virus (SARS-CoV2) counts up to date (November 2020) more than 55.6 million people infected and 1.34 million deaths, since the start of the pandemic. According to the latest knowledge, disease pathogenesis is driven by a dysregulated immune response against SARS-CoV2, characterized among others by impaired type I IFN production, sustained inflammation and aberrant cytokine production (2, 3) similar to the “cytokine storm” syndrome that can become life-threatening. The consequences of this hyper immuno-activation, lead eventually to the clinical manifestations of severe COVID-19 including respiratory failure, systemic inflammation, acute respiratory distress syndrome (ARDS), multi-system inflammatory syndrome and multi-organ fibrosis and malfunction (4).

It is currently believed that alveolar macrophages may lay in the center of COVID-19 pathogenesis and lethality. SARS-CoV2 infection in the lungs and failure of rapid virus clearance, leads to severe inflammation as well as, tissue damage and fibrosis. This prolonged overactivation of resident macrophages by SARS-CoV2 and augmented release of pro-inflammatory cytokines in the circulation, results in clinical manifestations similar to those described in the macrophage activation syndrome (MAS) (5). Therefore, identifying the factors that will regulate the macrophage responses in severe COVID-19 cases is essential for the disease recovery. In this review, we discuss the role of Specialized Pro-resolving lipid Mediators (SPMs) in driving the macrophages immuno-functions and thus regulating the macrophage-induced inflammation. Moreover, we address the molecular events that relate the lipid mediators with the disease pathology and discuss the clinical potentials of SPMs in COVID-19 treatment.

## Macrophages as Key Orchestrators of the Inflammatory Milieu During Coronaviruses Infections

Macrophages represent an immune cell type of the myeloid lineage with broad spectrum of functions including among others phagocytosis of pathogens and debris, secretion of reactive species and cytokines, as well as, matrix remodeling and tissue repair (6, 7). Although they are considered as part of the innate immune system, they are known to bridge an adaptive immune response *via* presentation of antigens to T cells and activation of the latter in the tissues, *via* expression of co-stimulatory molecules and secretion of cytokines (6, 7). Recent evidence is also suggestive of the existence of memory macrophage populations driven by epigenetic modifications events, described under the term “trained immunity” (8). These exact “innate memory” properties of macrophages are currently under investigation in COVID-19 immunotherapeutics (9). Traditionally, the functional heterogeneity of macrophages is represented *in vitro* by a rather oversimplified model, dividing macrophages into two main phenotypes (M1 and M2). M1 phenotype refers to pro-inflammatory or classical activated macrophages, associated with Th1 responses and IFNγ and/or LPS activation, while the M2 phenotype refers to anti-inflammatory or alternatively activated macrophages, associated with Th2 responses and IL-4 and/or IL-13 activation (10, 11). The terms “killing” or “healing” are also regularly used to describe M1 or M2 populations respectively. However, *in vivo*, there is not a clear cut off between these phenotypes but rather a continuum of functional states (11, 12) with M1 and M2 representing the opposing extremes of activation, thus delineating the need for reassessment of the current M1/M2 definition (13). Aiming for a *lingua franca*, a recent macrophage nomenclature has recommended the latest guidelines for macrophages activation, including the usage of the activator used *in vitro* instead of the typical M1 or M2 [e.g., M(IFNγ)] and the combinational employment of multiple membrane markers as well as, transcription factors for phenotypic characterization of macrophages subtypes (14).

Along with neutrophils, macrophages constitute the first line of immune defense against microbial or viral infections. Invader sensing *via* a vast variety of pattern recognition receptors (PRRs), including Toll-like receptors (TLRs), RIG-1 like receptors (RLRs) and NOD-like receptors (NLRs) (15), located either on the cell surface or in intracellular compartments, leads to macrophage phenotype shift and pro-inflammatory activation. The single strand RNA receptor TLR7 has been addressed as a sensor for SARS-CoV2 (16). Of note, loss of function studies of TLR7 showed correlation with disease severity and dysregulated IFN type I response (17). Activation of the RNA sensing receptors like the TLR7 and TLR8 triggers downstream signaling pathways like NF-κB which in turn leads to transcription of various pro-inflammatory cytokines e.g., IL-1β, TNFα, IL-6, known as the first cytokine wave [reviewed in (18)]. A delayed IFN type I response, either due to genetic reasons or *via* direct viral-induced immuno-suppressive mechanisms (19–22), leads to impaired virus control and increased accumulation of inflammatory monocytes and macrophages with negative effects on T cells, as shown in a mouse model of SARS-CoV infection (23). Paradoxically this hyperactivation was induced by the type I IFN *per se*, pointing to the importance of timing for an efficient clearance of the virus. In any case, an increasing viral load results in continuously elevating numbers of activated macrophages and sustained hyperinflammation—known as the second cytokine wave—with severe tissue damage and life-threatening consequences [reviewed in (18)]. Very recently, several molecules associated with macrophages functions, such as the plasma levels of long pentraxin 3 (PTX3), as well as, the ratio of the transcription factors MAFB/MAF have been suggested as potential prognostic indicators of disease progression and severity (24, 25). Of note, it has been speculated that the differences in disease severity between adults and children might be explained by the potential age-heterogeneity of macrophage populations in lungs and other organs during development (26) as well as, by the potential development of memory macrophages upon frequent adjuvant-involving vaccination during childhood (27).

Single-cell analysis has identified an FCN1^+^ inflammatory macrophage phenotype in critical COVID-19 cases expressing the pro-inflammatory mediators IL-1β, IL-8, TNF, CCL2, CCL3, CCL20, CXCL1, CXCL3, and CXCL10 (28). It is believed that this macrophage population actively regulates disease progression and fuels inflammation by continuously recruiting monocytes from the circulation and driving their differentiation. Same cytokines were also found in plasma from deceased COVID-19 patients (21). Similarly, the bronchoalveolar lavage fluid (BALF) from patients with severe COVID-19 was found rich in pro-inflammatory monocyte-derived macrophages (29). Moreover, lung macrophages from severe disease were found to produce higher levels of inflammatory cytokines (IL-6, IL-1β) as well as, monocyte and neutrophil chemoattractants (IL-8, CCL2, CCL3, CCL4, CCL7), whereas macrophages from moderate COVID-19 patients produced higher volumes of the T-cell chemokine CXCL16 (29). Indeed, hypercytokinemia is considered a hallmark of the disease, while the serum levels can differentiate the disease severity (30). The direct implication of the cytokines involved in disease pathophysiology and the associated pneumonia, found both in MAS and in COVID-19, is highlighted in preliminary trials against SARS-CoV2, where blockade of IL-6 with tocilizumab showed promising clinical efficacy (31). However, whether IL-6 is detrimental or beneficial in COVID-19 remains unclear and is highly probable that timing is the most important factor in determining the success or not of anti-IL-6 treatments. Blockade of IL-6 at the early stages of the disease development might negatively affect effective virus clearance [reviewed in (32)].

To this extent, treating the inflammation and the cytokine storm has emerged as a legitimate approach in COVID-19 therapeutics and appears to be equally important to anti-viral therapies. Various strategies have been proposed in this vein, including cytokine inhibitors (e.g., tocilizumab), corticosteroids, intravenous immunoglobulin (IVIG) and cytokine absorption devices [reviewed in (18, 33)]. Since macrophages may also stand out as the key cell population in the resolution of inflammation and healing of the damaged tissue, identifying the factors inducing their phenotypic shift from the ‘‘pathologic’’ phenotype to the ‘‘healing’’ one, is essential in COVID-19.

## SPMs in Inflammation and COVID-19

Specialized pro-resolving mediators **(**SPMs)—consisting of lipoxins, resolvins, maresins, and protectins—represent a novel class of bioactive lipids that are generated by enzymatic oxygenation of n-3 and n-6 polyunsaturated fatty acids (PUFAs) after the initial stages of the inflammatory cascade (34). Endogenous biosynthesis of SPMs involves cell–cell interactions and is mediated by lipoxygenases (35). As the acute inflammatory response matures, accumulation of cells containing lipoxygenases (LOs) and corresponding pro-inflammatory products, such as prostaglandins (PGs), leukotrienes (LTs) and hydroxy acids (HETES), favors the ‘‘lipid mediator class switching’’. This phenomenon, as was initially described in neutrophils gives rise to the synthesis of SPMs through pathways that are spatially and temporally distinct from those involved in the generation of pro-inflammatory lipid mediators (36, 37). Lipoxins are synthesized through a series of enzymatic reactions starting with the oxidation of arachidonic acid (AA) by 15-LO through the process of transcellular biosynthesis, resulting in 15-S-hyroxy-(p)-eicosatetraenoic acid' [15-S-H(p)ETE]. Accordingly, 15-S-H(p)ETE is further acted on by 5-LO to generate lipoxins, such as lipoxins A_4_ (LXA_4_) and B_4_ (LXB_4_) (36, 37). Similar effects can be reproduced by exogenous administration of low-dose aspirin, which acetylates cyclooxygenase-2 *via* 15R-LO, promoting the biosynthesis of epimeric (aspirin-triggered) (AT) forms of SPMs (15-epi-LXs or ATLs) (38).

E-series resolvins are produced by vascular endothelium *via* aspirin-modified COX-2 that converts eicosapentaenoic acid (EPA) to 18R-hydro-peroxyeicosapentaenoic acid (18R-HEPE) and 18S- hydro-peroxyeicosapentaenoic acid (18S-HEPE). These intermediates are rapidly taken up by human neutrophils and are metabolized to resolvin E1 (RvE1) and RvE2 by 5-LO. RvE1 biosynthesis can also be initiated by microbial cytochrome P450 mono-oxygenase in an aspirin-independent manner, which can contribute to its production *in vivo* (39, 40). D-series resolvins and protectins derive from docosahexaenoic acid (DHA) *via* subsequent 15-LO and 5-LO-mediated actions, while maresins are generated through 12-LO-mediated pathways (41). Thus, even though qualitatively the same enzymes are involved in either SPMs or eicosanoid biosynthesis, the exact molecular and biochemical mechanisms driving temporal relationships during the lipid mediator class switching remain to be further elucidated.

The balance of PUFAs-derived mediators in leukocytes has been associated with the localization of 5-LO (42). In fact, studies on macrophages in heightened inflammatory lesions such as advanced atherosclerotic plaques described abundant nuclear localization of 5-LO. Nuclear 5-LO because of its proximity to LTA4 hydrolase, seems to promote the conversion of AA to pro-inflammatory LTs in macrophages (43). On the contrary, non-nuclear localization of 5-LO, possibly due to its proximity to 12/15-LO, may favor the conversion of AA or DHA to lipoxins or D-series resolvins respectively and has been therefore linked with enhanced SPMs formation (42–44). Interestingly, RvD1 has been described to shift 5-LO from the nucleus to the cytoplasm inducing a negative feedback loop that suppresses LTB_4_ formation and promotes generation of LXA_4_ in macrophages (44). In addition to the relocalization events, lipid mediator biosynthesis may be also affected by post-translational modifications, including miRNAs. For instance, increased expression of miR-466I in macrophages at early stages of inflammation promoted the synthesis of RvD1 in macrophages and the resolution of inflammatory exudates in mice (45). The regulation of resolution of inflammation by miRNAs *via* increasing the synthesis of SPMs and mediating resolution-phase macrophage polarizations needs to be further investigated. Once generated, SPMs act as agonists at specific G-protein-coupled receptors expressed on various cells including monocytes and macrophages and activate the resolution of inflammation, without causing systemic immunosuppression (46).

Among these receptors is ALX/FPR2, which apart from SPMs binds also protein ligands, such as, the acute-phase protein serum amyloid A (SAA) (47). Recently, SAA plasma levels were found to be dynamically increased with COVID-19 disease severity and this protein has been therefore proposed as a biomarker indicative of the severity and prognosis of the disease (48). Of note, SAA can antagonize the signaling of LXA_4_ and *vice versa* through allosteric inhibition of the receptor, inducing opposite intracellular effects (47). In fact, SAA inhibited the LXA_4_-mediated protective signaling in patients with chronic obstructive pulmonary disease resulting in defected activation of anti-inflammatory circuits providing a molecular explanation for SAA-mediated impaired resolution (49).

Apart from SAA overexpression, COVID-19 has been characterized by elevated production of macrophage-derived eicosanoids that further enhance inflammation (50). In addition, a recent lipidomic analysis study showed a significant shift in the profile of lipid mediators with increased levels of arachidonate-derived proinflammatory lipid mediators (prostaglandins) in the sera of COVID-19 patients (51). Grouping of lipid mediators according to the oxygenase-mediated synthesis pathway, demonstrated a greater activity of ALOX5, ALOX15, and cytochrome p450 (CYP) enzymes in the severe group (51). Further examination on the cell types responsible for the addressed lipidomic imbalance in this severe COVID-19 group, alluded neutrophils, as well as, a trend for CD14+, and CD16+ monocytes. Elevated ALOX5 expressing monocyte/macrophage populations have also been associated with severe disease (51). Similarly, increased ALOX5 activity related to symptom severity has been addressed in other viral infections e.g., in influenza (52).

Recently, COVID-19 has been also linked to dyslipidemia associated with deficiency of apolipoprotein E (ApoE) (50). ApoE is generated among other cells by lung macrophages and alveolar epithelial cells and exerts its protective effects by downregulating VCAM‐1, inducing NO synthesis, inhibiting endothelial activation, and decreasing adhesion of monocytes to the endothelium (53). ApoE^−/−^ mice under an omega-3 fatty acid-deficient diet presented significant overexpression of pro-inflammatory eicosanoids in the lungs as well as endothelial dysfunction which can contribute to increased blood coagulation found in severe COVID-19 (54). It has been therefore suggested that deficiency in ApoE found in SARS-CoV2 dyslipidemia may be linked to disease progression and complications (50). Of note, supplementation with EPA and DHA downregulated the levels of pro-inflammatory eicosanoids such as thromboxane B2 in the lungs of ApoE^−/−^ mice and the effect was even more pronounced with the addition of aspirin treatment (54).

Known risk factors for severe COVID-19, including diabetes, obesity and chronic obstructive pulmonary disease (COPD), have been also related to dysregulated concentrations of SPMs (55, 56). Collectively these findings underscore the importance of fine-tuned lipid mediators’ responses to achieve resolution in inflammation-associated pathologies. Severe COVID-19 may be characterized among others by a lipidomic imbalance in key cell populations contributing to the disease progression, such as neutrophils and/or monocytes/macrophages. As such, understanding the full anti-inflammatory and pro-resolving spectrum of these lipid mediators is of major importance in the current coronaviruses pandemics.

## SPMs in Regulating Macrophage Immuno-Functions: Macrophage Infiltration and Mobility

Recent evidence suggests that severe clinical manifestations found in COVID-19 are associated with excessive infiltration of inflammatory monocytes in the lungs, in the expense of tissue-resident alveolar macrophages (29) and the subsequent release of pro-inflammatory cytokines in the circulation (5). Moreover, a population of these infiltrated monocyte-derived macrophages may be associated with the pulmonary fibrosis (57, 58), also found in progressed COVID-19 cases. Following a gradient of chemoattractant molecules and cytokines, circulating monocytes massively infiltrate the lungs, kidneys and other organs. In this vein, SPMs have been shown to inhibit monocyte recruitment into the tissues by regulating the leukocyte-endothelial interactions. Exogenous addition of resolvin E1 (RvE1) in whole blood was found to downregulate monocyte surface expression of adhesion molecules, mediating both early activation and rolling (i.e. L-selectin), as well as subsequent stable adhesion on the endothelium and transmigration into the tissues (e.g., CD18 integrin) (59). Incubation with RvE1 reduced the migration of M1-like macrophages (activated with LPS) towards the chemotactic protein chemerin, concomitant with a downregulation in the expression of ChemR23 receptor on macrophage surface, pointing to the regulatory role of this receptor in migration of macrophages to the inflamed area (60). Local application with RvE1 also decreased the numbers of infiltrating neutrophils, as well as, Th1 and Th17 cells in the cornea of mice with HSV-1-induced stromal keratitis (61). Similarly, other SPMs (LXA_4_ and RvD1), in solution or incorporated into nano-proresolving medicines, reduced monocyte trafficking toward LTB_4_ (62). Of note, in a bacterial and viral lung co-infection model with robust recruitment of infiltrating monocytes and increased counts of exudative macrophages, exogenous delivery of AT-RvD1 during the acute phase of infection (day 4–6 post-pneumococcal inoculation), resulted in ∼50% reduction in infiltrating monocytes/macrophage numbers (CD11b^Hi^, CD11c^Low^) (63). Since transcript or protein levels of key cytokines and chemokines associated with monocyte recruitment such as IL-1β, IL-6, CCL2 and monocyte chemoattractant protein-1 (MCP-1) were not significantly reduced by this SPM, the authors suggested that AT-RvD1 directly affected the chemotactic properties of inflammatory monocytes (from the infected bronchioles to the distal lung alveoli) *per se* (63). In addition, halting further monocyte recruitment could prevent a monocyte-induced secondary phase of neutrophil migration into the lungs that would perpetuate acute lung injury. Interestingly, treatment with AT-RvD1 did not reduce exudative macrophages (CD11b^Hi^, CD11c^Hi^) (63), a subpopulation of particular importance in the resolution processes represented by their ability to clear apoptotic neutrophils and facilitate the return to tissue homeostasis (64). Thus, SPMs may hold promising therapeutic potentials in decelerating the pathologic macrophage infiltration in inflamed lungs, during COVID-19 development.

## SPMs in Regulating the Cytokine/Chemokine Expression of Monocytes/Macrophages

A growing body of evidence points to the regulatory role of SPMs in pro-inflammatory cytokine secretion by a variety of immune cells, including monocytes and macrophages. SPMs inhibit the secretion of TNF, IL-1β, IL-8 by activating the GSK3β anti-inflammatory axis in LPS-stimulated primary human monocytes (65). Resolvins inhibit the production of TNF-α and IL-6 by alveolar macrophages derived from both COPD and non-COPD individuals, although with a stronger potency in the latter (56). In addition, AT forms of SPMs were shown to decrease the secretion of macrophage migration inhibitory factor (MIF), plasminogen activator inhibitor-1 (PAI-1) and chemokine CCL2 in human monocyte-derived macrophages (66). Research focus on SPMs has recently resulted in the identification of novel peptide-containing conjugates with docosahexaenoic acid (DHA)-derived backbones. Resolvin peptide-containing conjugates in tissue regeneration (RCTRs), down-regulate inflammatory chemokines CXCL9 and CCL7 and increase IL-10 in human macrophages (67). Thus, in addition to the downregulation of pro-inflammatory molecules, SPMs and SPMs-peptide conjugates stimulate the production of anti-inflammatory cytokines by macrophages such as IL-10 and TGF-β (56), as well as of molecules associated with alternative macrophage activation (M2) and tissue healing e.g., IL-4, IL-11, and TGF-α (67). Physiologically, human macrophages may respond to different pathogens differently, as regards the lipid mediators’ synthesis, depending on their activation phenotype. It was shown that *Escherichia coli* and *Staphylococcus aureus* stimulated the production of LTB_4_ and PGE_2_ in M1 macrophages, while favored the secretion of SPMs, including RvD2, RvD5 and maresin-1 in M2 macrophages (68). Mechanistically, SPMs act by regulating a variety of downstream signaling pathways including: both canonical and alternative NF-kB pathways, STAT3, cAMP response element binding protein and MAPK signaling pathways (56, 69). *In vivo* evidence is also not lacking; RvD1 downregulates various pro-inflammatory cytokines and chemokines including TNF-α, IL-6, CCL2, and IL-1β in peritoneal macrophages in mice (70). Of note, the involvement of macrophages in COVID-19 and the therapeutic potential of SPMs in the disease is further highlighted in a recent study; short incubation (3h) of monocyte-derived macrophages isolated from cystic fibrosis (CF) individuals with S1, S2 and N proteins from SARS-CoV-2 resulted in rapid release of chemokines such as IL-8 in cell-free supernatants (71). Most importantly, treatment with SPMs of CF macrophages stimulated with S1 protein resulted in a significant reduction in the release of IL-8, MCP-1 and macrophage inflammatory protein MIP-1α (CCL3) and MIP-1β (CCL4) (71). Thus, treating the macrophage-generated cytokine storm in severe COVID-19 with SPMs, has emerged as a legitimate approach.

## SPMs in Regulating the Macrophage-Mediated Efferocytosis and Thrombus Resolution

As noted above, clearance of dead cells and debris by professional phagocytes in the inflamed area—following the removal of the pathogen—is essential part of the resolution process. In fact, the uptake of dead cells (efferocytosis) per *se* or the extracellular vesicles secreted by dead cells, drive the reprogramming of macrophages towards an anti-inflammatory state (72–74). Macrophages play a critical role in orchestrating infectious inflammation towards its resolution (68). Accumulating evidence has documented a significant increase in macrophage uptake of apoptotic cells, following *in vitro* stimulation with SPMs, underlining their role as potent enhancers of macrophage efferocytosis (75, 76). Inflammation is characterized by a hypoxic milieu that develops, in part, *via* increased oxygen consumption by infiltrating leukocytes (77). SPMs promoted macrophage efferocytosis of neutrophils and erythrocytes in hemorrhagic exudates under hypoxic conditions *in vivo*. Of note, the engulfment of apoptotic PMNs and PMN microparticles by macrophages during efferocytosis increases the autocrine SPMs biosynthesis (78). In this vein, uptake of apoptotic cells by M1 macrophages (activated with IFNγ+LPS) induced the production of SPMs, while suppressed prostanoids and leukotrienes, suggesting the presence of an endogenous negative feedback loop (79). In addition, aspirin-triggered forms of SPMs identified in patients with coronary artery disease receiving n-3 PUFA supplementation, significantly upregulated macrophage phagocytosis of blood clots, thereby promoting clot removal (80). Given the high risk for thrombosis found in severe COVID-19 this may represent an additional protective function of SPMs related to their regulatory role on macrophages. Moreover, SARS-CoV2 was recently shown to stimulate the formation of neutrophil extracellular traps (NETs) in human neutrophils. The activation and release of NETs (NETosis) was found to be associated with increased levels of intracellular Reactive Oxygen Species (ROS) in these cells and was suggested to play an important role in thrombosis formation in COVID-19 patients (81). Interestingly, *in vivo* treatment with SPMs in mice was recently described to inhibit NETosis, indicating another protective effect of SPMs in thrombus resolution that could be of particular importance for COVID-19 (82, 83). SPMs may therefore have a beneficial effect on different cell types against COVID-19, towards the removal of dead cells and blood clots, as well as the resolution of inflammation and thrombroinflammation.

## SPMs in Regulating the Macrophage Polarization

Given the accumulating number of inflammatory macrophages infiltrating into lungs and other tissues and the natural plasticity of macrophages, a phenotypic shift of this pathogenic population may hold strong therapeutic potentials in COVID-19. Recent findings underlined the potential of SPMs to induce changes in macrophage phenotype towards a resolving profile, as indicated by a significant downregulation in the expression of M1-associated markers (CD54 and CD80) concomitantly with an increase in M2-associated ones (CD163 and CD206) (84). Along these lines, RvD2 prevents cigarette smoke extract (CSE)-induced M1 polarization (CD80) and enhance M2 polarization (CD206) in monocyte-derived macrophages (56). Further evidence is suggestive for the existence of distinct lipid mediator profiles among different macrophage subtypes; M2 macrophages (CD163 and CD206) have been found to generate higher levels of SPMs, whereas M1 macrophages (CD68 and CD86) have been shown to produce elevated amounts of prostanoids and leukotrienes, thereby promoting the amplification of inflammation (85). Moreover, the activating protein of ALOX5 enzyme was induced in M1, but not in M2 macrophages, while ALOX15 was upregulated in M2, but not in M1, macrophages (85). It was recently found that macrophages produce also a family of DHA-derived SPM molecules, named maresins (MaRs) with autocrine actions (79). *In vitro* treatment with the bioactive SPM, 13,14-epoxy-MaR resulted in higher levels of MaR1 in M2 macrophages (CD163 and CD206) compared to M1 macrophages (CD54 and CD80), suggesting a higher predisposition towards the proresolving activity of this macrophage subtype (84). Remarkably, *in vivo* administration of RvD2 favored a macrophage phenotype (Ly6C^lo^), with unique pro-resolving properties, stimulating inflammation resolution and tissue (muscle) regeneration (86).

A key phenotypic characteristic of mouse pro-inflammatory macrophages (M1) is the excessive production of nitric oxide (NO) (10, 14) and other reactive species, as part of their oxidative burst anti-microbial mechanisms. Maresin-like lipid mediator 14S,21R-dihydroxy-docosahexaenoic acid was shown to reduce the hyperglycemia-induced ROS production by macrophages (87) and modulate the ability of mesenchymal stem cells to induce ROS generation from macrophages under ischemia/reperfusion conditions (88). LXs and their aspirin-triggered epimers were shown to interfere with reactive species production by various cell types including monocytes and neutrophils (89, 90). Given the potential relationship of oxidative stress with the pathogenesis of COVID-19 (91), SPMs may have further beneficial effects in the current pandemic.

Thus, and in line with the previous functional effects mentioned above, a growing body of evidence points to the SPMs-induced phenotypic shift of macrophages towards an anti-inflammatory and pro-resolving subtype.

## SPMs in Regulating the Viral Cell-Infection and Tissue Fibrosis

Apart from their regulatory role in monocytes and macrophages, SPMs may also exhibit direct anti-viral effects, further enhancing their therapeutic potentials in complementing current anti-viral strategies. Stimulation of isolated macrophages from volunteers with CF with S1 protein from SARS-CoV2 triggered the biosynthesis of RvD1 pointing to the activation of pro-resolving signals in response to SARS-CoV2 (71). Activation of TLR7, an important pattern recognition receptor of viral RNA as noted above, is also known to stimulate SPMs production (92). Of note, a direct mechanistic effect of SPMs was also shown; protectin 1 interacts with the RNA replication machinery of influenza virus and inhibits viral RNA nuclear export, thereby suppressing the pathogenicity of the virus (93). Although similar protective events have not yet addressed for SARS-CoV2, given the similarities in the replication of RNA viruses, one cannot exclude the high possibility of such speculation. Of note, 17-HDHA has been shown to increase antibody production (IgM, IgG) by enhancing B cell differentiation towards an antibody-secreting B cell phenotype (CD80, CD86) in H1N1 influenza infection in mice (94) alluding to the regulatory effects of SPMs in the humoral immune response against viruses.

As noted above, advanced COVID-19 is also characterized by excessive fibrosis in lungs and other organs, most probably as a consequence of failures in the regulation of inflammation; unresolved inflammation has been closely related to tissue fibrosis and impaired organ function (95). Proinflammatory eicosanoids, such as leukotrienes, promote tissue fibrosis, whereas AT-LXs and synthetic benzo-LXA_4_ analog have been shown to reduce bleomycin-induced pulmonary fibrosis and renal fibrosis respectively (96, 97). SPMs protective effect against fibrosis, is mediated at least in part by the reduction in collagen deposition (97). Thus, SPMs may also hold additional therapeutic modalities against severe COVID-19 manifestations, such as multiple organ fibrosis.

## Conclusions and Perspectives

**Figure 1:**
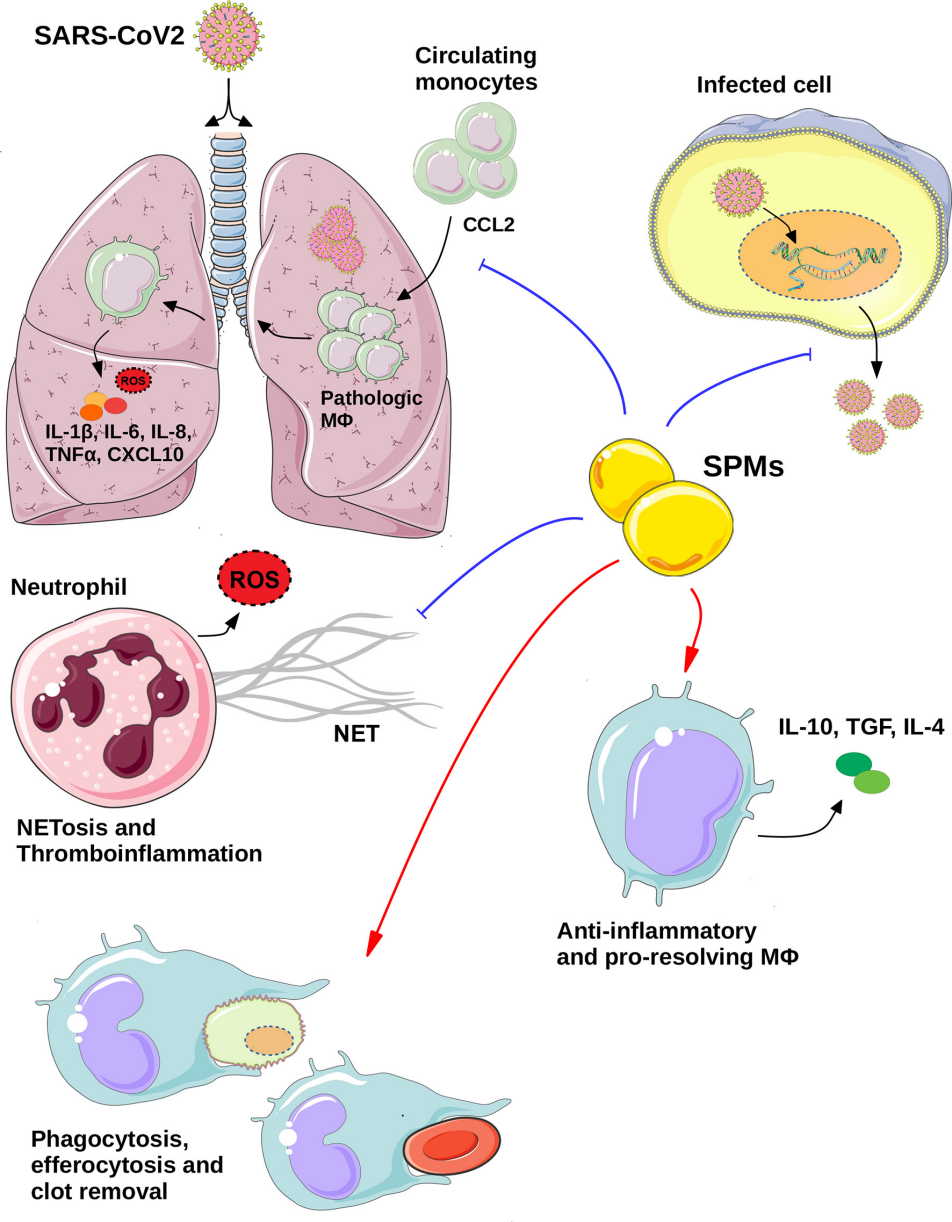
The potential therapeutic role of SPMs in COVID-19. Specialized pro-resolving regulators (SPMs) mediate their anti-viral effects *via* directly inhibiting the viral replication and thus export of viral particles from infected cells and/or *via* regulating the functions of key immune cell types. SPMs act on neutrophils inhibiting NETosis and thrombroinflammation, while they regulate the macrophages responses on various levels including: inhibition of monocytes infiltration to the inflammed lungs, as well as, stimulation of homeostatic processes, *via* induction of efferocytosis, phagocytosis of debris and blood clots and anti-inflammatory and pro-resolving macrophages polarization. All these effects may eventually lead to the resolution of SARS-CoV2-induced inflammation and thus, potential management of COVID-19 pathologies. Blue lines indicate inhibition, whereas red arrows indicate induction. (Templates from Servier Medical ART, https://smart.servier.com/, were used to create this image) MΦ, Macrophage; ROS, Reactive oxygen species.

Corona virus pandemic of 2019 has emerged as one of the biggest threats of our times, with outrageous health, social, economic and financial consequences globally. Since the identification of a cytokine storm in severe COVID-19 and the central role of pathogenic alveolar macrophage populations in the maintenance of hyperinflammation, characterized the severe disease, many current therapeutic research efforts have focused on anti-inflammatory approaches. However, treating individual cytokines may not be sufficient. SPMs have the advantage of stimulating the physiological homeostatic processes towards the resolution of inflammation, acting directly on macrophages on various levels (**Figure 1**), without inducing systemic immuno-suppression. Moreover, clinical experimentation has alluded to the safety of SPMs in humans. Exogenous administration of EPA and DHA (Lovaza) restored the endogenous SPMs levels in coronary artery patients, without any severe adverse effects being reported (80). Furthermore, SPMs may also hold beneficial effects against COVID-19, beyond regulating macrophages responses. **Table 1** summarizes the immuno-regulatory functions of SPMs in relation to disease pathology, as discussed here. Interspecies differences in macrophage functions may well exist [discussed in (98)]; however many of the studies on SPMs addressed here, have used human primary macrophages. The importance of lipid mediators in COVID-19 progression is highlighted by studies showing a direct link between a lipidomic imbalance and disease severity. Even though SPMs are excessively attractive as potential therapeutic candidates in COVID-19 (99–102), either exogenously administered or by inducing their endogenous production in patients, the relevant clinical evidence is still lacking, yet highly anticipated.

**Table 1:** Immunoprotective actions of SPMs in relation to COVID-19 pathology.

SPM	Cell-type/disease model	Mechanism of action	Reference
**Leukocytes adhesion, chemotaxis and migration**			
Resolvin E1	Human whole blood, intravital microscopy in mice venules	Downregulates leukocyte expression of adhesion molecules Reduces leukocyte rolling (∼ 40%) on the endothelium	Dona et al. (59)
AT- Resolvin D1	Bacterial and viral lung co-infection model in mice	Reduces infiltrating monocytes/macrophage numbers (CD11b^Hi^, CD11c^Low^)	Wang et al. (63)
Resolvin E1	Human M1-like macrophages	Reduces migration of M1-like macrophages (activated with LPS) towards the chemotactic protein chemerin	Herova et al. (60)
Resolvin D1 Lipoxin A_4_	*In vitro* microfluidic inflammation model based on primary human neutrophil and monocytes	Suppress monocyte trafficking toward LTB_4_	Jones et al. (62)
**Generation of cytokines and chemokines**			
Resolvin D1 Resolvin D2	Human macrophages from cystic fibrosis individuals stimulated with S1 protein from SARS-CoV2	Reduce release of IL-8, MCP-1, MIP-1α and CCL4 Upregulates expression of IL1RA	Recchiuti et al. (71)
AT- Resolvin D3 AT-Lipoxin A_4_	Human monocyte-derived macrophages	Decrease secretion of MIF, PAI-1 and CCL2	Gilligan et al. (66)
Resolvin D1 Resolvin D2 Maresin 1	LPS-stimulated primary human monocytes	Inhibit secretion of TNF-α, IL-1β, IL-8 by activating the GSK3β anti-inflammatory axis	Gu et al. (65)
Resolvin D2	Alveolar macrophages from COPD and non-COPD individuals	Downregulate production of TNF-α and IL-6, stimulate the production of IL-10 and TGF-β	Croasdell et al. (56)
Resolvin peptide-containing conjugates in tissue regeneration (RCTRs)	Human monocyte-derived macrophages	Downregulate CXCL9 and CCL7 Increase IL-10, IL-4, IL-11 and TGF-α	De la Rosa et al. (67)
Resolvin D1	Peritoneal macrophages in mice	Decreases TNF-α, IL-6, CCL2 and IL-1β	Kain et al. (70)
**Phagocytosis and efferocytosis**			
Resolvin peptide-containing conjugates in tissue regeneration (RCTRs)	Human monocyte-derived macrophages	Stimulate phagocytosis and efferocytosis	De la Rosa et al. (67)
Resolvin D2	Alveolar macrophages from COPD and non-COPD individuals	Restore cigarette smoke-induced defects in phagocytosis	Croasdell et al. (56)
AT- Resolvin D3 Resolvin D6 AT-Protectin D1 AT-Lipoxin B_4_	Human monocyte-derived macrophages	Upregulate macrophage phagocytosis of blood clots and promote blood clot removal	Elajami et al. (80)
**Macrophages polarization**			
Maresin 1 Resolvin D1	Human monocyte-derived macrophages	Downregulate M1-associated markers (CD54 and CD80) and increase M2-associated markers (CD163 and CD206)	Dalli et al. (84)
Resolvin D2	Human monocyte-derived macrophages	Prevents cigarette smoke extract (CSE)-induced M1 polarization (CD80) and enhance M2 polarization (CD206)	Croasdell et al. (56)
13,14-epoxy-Maresin	Human monocyte-derived macrophages	Increases the levels of SPMs in M2 macrophages (CD163 and CD206)	Dalli et al. (84)
Resolvin D2	Muscle-infiltrating macrophages from mice	Increases Ly6C^lo^ macrophages and promotes regeneration	Giannakis et al. (86)
**NETosis and thrombus resolution**			
Resolvin D1	Abdominal aortic aneurysm model in mice	Suppresses NETosis markers, IFNγ, IL-1β, CXCL10 and MCP-1	Spinosa et al. (82)
Resolvin D4	Deep vein thrombosis model in mice	Inhibits ionomycin-induced release of NETs and promotes thrombus resolution	Cherpokova et al. (83)
**Oxidative stress**			
14S,21R-dihydroxy-docosahexaenoic acid	Murine macrophages isolated by peritoneal lavage	Improves diabetes-impaired pro-healing functions of macrophages by reducing hyperglycaemia-induced ROS production	Tian et al. (87)
14S,21R-dihydroxy-docosahexaenoic acid Lipoxin A4 AT 15-epi-LXA4	Murine macrophage cell line (RAW264.7)	Modulate the ability of mesenchymal stem cells to induce ROS generation from macrophages under ischemia/reperfusion conditions	Tian et al. (88)
	Human neutrophils and monocytes	Reduce superoxide and peroxynitrite (ONOO−) formation Inhibit increase in intracellular diamino-fluorescein fluorescence (indicator of NO formation)	József et al. (90)
**Anti-viral effects**			
Protectin 1	Influenza in mice	Interacts with the RNA replication machinery of influenza virus, inhibits viral RNA nuclear export and improves the survival	Morita et al. (93)
17-HDHA	H1N1 influenza infection in mice	Increase the number of antibody-secreting cells *in vitro* protecting against live pH1N1 influenza infection	Ramon et al. (94)
AT- Resolvin D1	Co-infection with *Streptococcus pneumoniae* and influenza A virus in mice	Reduces severity of pneumonia by inhibiting excessive leukocyte chemotaxis from the infected bronchioles to distal areas of the lungs	Wang et al. (63)
Resolvin E1	HSV-1-induced stromal keratitis (SK) in mice	Reduce the number of Th1,Th17 cells and neutrophils infiltrating the cornea	Rajasagi et al. (61)
**Anti-fibrotic effects**			
AT-synthetic lipoxin analog	Pulmonary fibrosis in mice	Reduces inflammation and matrix deposition Inhibits bleomycin-induced pulmonary fibrosis and renal fibrosis	Martins et al. (96)
Lipoxin A4 synthetic benzo- Lipoxin A4	Early renal fibrosis in mice	Decreases collagen deposition and inhibits progression of renal fibrosis	Börgeson et al. (97)

## Author Contributions

MB performed the literature research and wrote the manuscript. EP contributed to manuscript writing and formatting and provided critical scientific input. PC conceived the idea, designed the lay out, and wrote and reviewed the manuscript. All authors contributed to the article and approved the submitted version.

## Funding

This research was supported by funds provided by the South-Eastern Norway Regional Health Authority, Project Number 2019059 to PC. Supported in part by USPHS grant K08DE027119 to EP from the National Institute of Dental and Craniofacial Research (NIDCR).

## Conflict of Interest

The authors declare that the research was conducted in the absence of any commercial or financial relationships that could be construed as a potential conflict of interest.

## Abbreviations

ALOX5, 5-lipoxygenase; ALOX15, 15-lipoxygenase; ARDS, Acute respiratory distress syndrome; (AT)-Rv, aspirin-triggered resolving; CCL, Chemokine (C-C motif) ligand; COVID-19, Corona virus disease 2019; CXCL, Chemokine (C-X-C motif) ligand; DHA, Docosahexaenoic acid; EPA, Eicosapentaenoic acid; IFN, Interferon; IL, Interleukin; LPS, Lipopolysaccharide; LTB_4_, Leukotriene B4; MaR, Maresin; MAS, Macrophage activation syndrome; NET, Neutrophil extracellular trap; NF-κB, Nuclear factor-κB; NLRs, NOD-like receptors; PMN, Polymorphonuclear leukocyte; PRRs, Pattern recognition receptors; PTX3, Long pentraxin 3; PUFAs, Polyunsaturated fatty acids; Rv, Resolvin; RLRs, RIG-1 like receptors; SARS-CoV2, Severe acute respiratory syndrome corona virus; SPMs, Specialized pro-resolving mediators; TLRs, Toll-like receptors; TNFα, Tumor necrosis factor α.
